# Sleep and circadian phenotype in people without cone-mediated vision: a case series of five *CNGB3* and two *CNGA3* patients

**DOI:** 10.1093/braincomms/fcab159

**Published:** 2021-07-18

**Authors:** Manuel Spitschan, Corrado Garbazza, Susanne Kohl, Christian Cajochen

**Affiliations:** 1 Department of Experimental Psychology, University of Oxford, Oxford, OX2 6GG, UK; 2 Centre for Chronobiology, Psychiatry Hospital of the University of Basel (UPK), CH-4002 Basel, Switzerland; 3 Transfaculty Research Platform Molecular and Cognitive Neurosciences (MCN), University of Basel, CH-4055 Basel, Switzerland; 4 Institute for Ophthalmic Research, Centre for Ophthalmology, University of Tübingen, D-72076 Tübingen, Germany

**Keywords:** circadian rhythms, sleep, congenital achromatopsia, rod monochromacy, dim-light melatonin onset

## Abstract

Light exposure entrains the circadian clock through the intrinsically photosensitive retinal ganglion cells, which sense light in addition to the cone and rod photoreceptors. In congenital achromatopsia (prevalence 1:30–50 000), the cone system is non-functional, resulting in severe light avoidance and photophobia at daytime light levels. How this condition affects circadian and neuroendocrine responses to light is not known. In this case series of genetically confirmed congenital achromatopsia patients (*n* = 7; age 30–72 years; 6 women, 1 male), we examined survey-assessed sleep/circadian phenotype, self-reported visual function, sensitivity to light and use of spectral filters that modify chronic light exposure. In all but one patient, we measured rest-activity cycles using actigraphy over 3 weeks and measured the melatonin phase angle of entrainment using the dim-light melatonin onset. Owing to their light sensitivity, congenital achromatopsia patients used filters to reduce retinal illumination. Thus, congenital achromatopsia patients experienced severely attenuated light exposure. In aggregate, we found a tendency to a late chronotype. We found regular rest-activity patterns in all patients and normal phase angles of entrainment in participants with a measurable dim-light melatonin onset. Our results reveal that a functional cone system and exposure to daytime light intensities are not necessary for regular behavioural and hormonal entrainment, even when survey-assessed sleep and circadian phenotype indicated a tendency for a late chronotype and sleep problems in our congenital achromatopsia cohort.

## Teaser

In this case series, Spitschan et al. investigate self-reported sleep and circadian phenotype, rest-activity cycles and melatonin production in a group of genetically confirmed congenital achromats (ACHM).

## Graphical Abstract

**Graphical Abstract:**
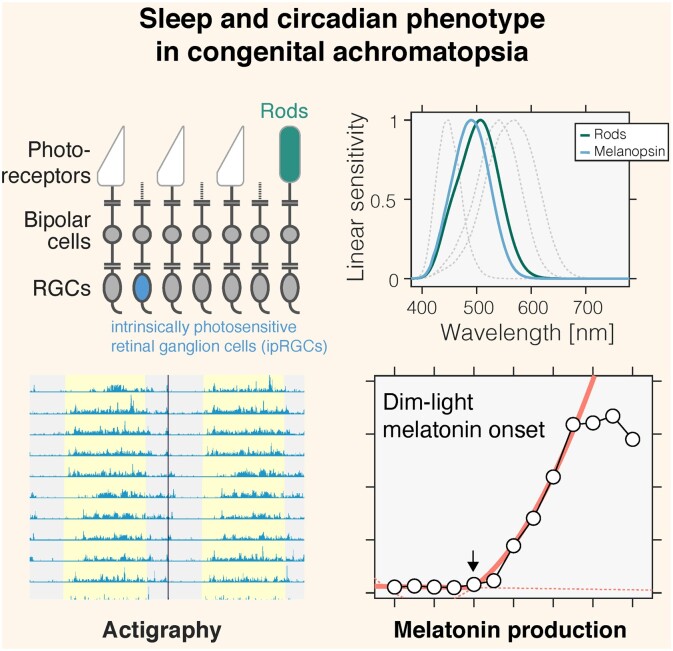

## Introduction

**Figure 1:**
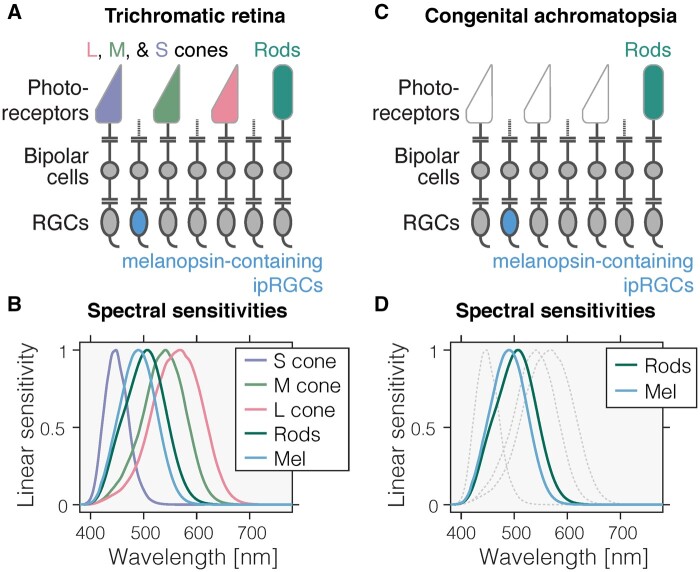
**Photoreceptors in the trichromatic and achromatic human retina.** (**A**) Schematic diagram of the normal, trichromatic human retina containing three classes of cones—long [L]-, medium [M]- - and short [S]-wavelength-sensitive cones—, rods, and the intrinsically photosensitive retinal ganglion cells (ipRGCs) expressing the photopigment melanopsin. (**B**) Spectral sensitivities of the photoreceptors in the trichromatic retina, showing the overlapping *in vivo* wavelength sensitivity for the S (λ_max_ = 448 nm in linear energy units after pre-receptoral filtering), M (λ_max_ = 541 nm), and L (λ_max_ = 569 nm) cones, the rods (λ_max_ = 507 nm), and melanopsin (λ_max_ = 490 nm). Spectral sensitivities shown here assume a 32-year-old observer and include pre-receptoral filtering.^85^ (**C**) Schematic diagram of the retina of a congenital achromat, missing functional cones, thereby only containing rods and ipRGCs. (**D**) Spectral sensitivities of the photoreceptors in the achromat retina. Faint dashed lines corresponding to the L, M and S spectral sensitivities are given for reference only.

**Figure 2:**
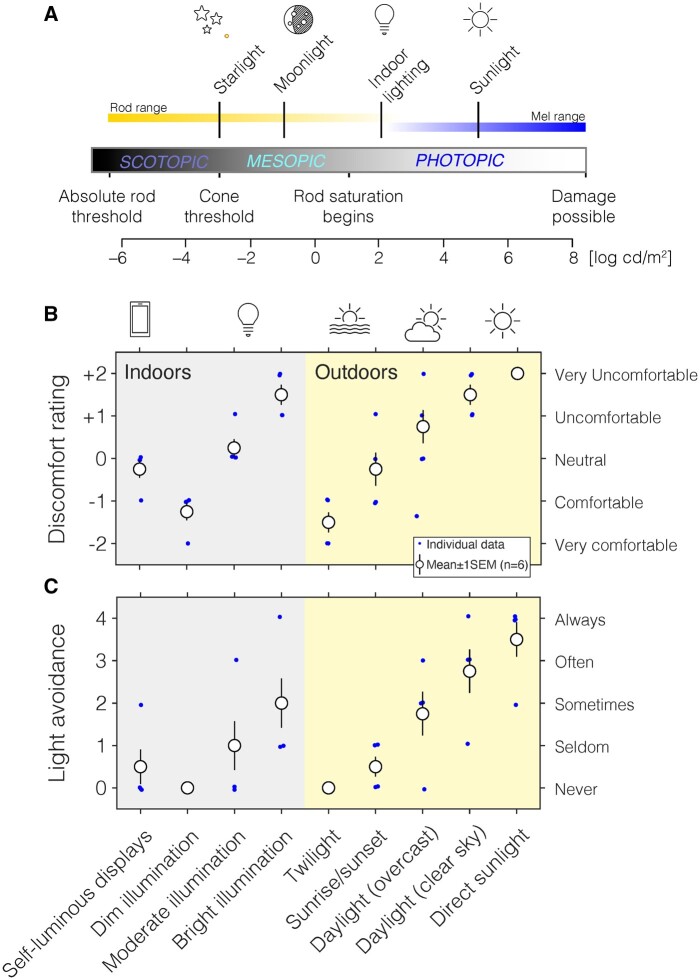
**Light sensitivity and light avoidance in congenital ACHM.** (**A**) Range of light levels and corresponding environmental conditions. The estimated, rod-based range of congenital achromats is indicated as a yellow, fading horizontal bar. We show the melanopsin operating range based on estimates by Dacey et al.^6^ (**B)** Ratings of light sensitivity and visual discomfort across a range of commonly encountered lighting conditions, indicating severe light sensitivity in bright light. (**C**) Ratings of light avoidance when filters are not used. To manage the hypersensitivity to light, congenital achromats use a range of filters that reduce retinal illumination. Individual data points are shown as blue dots, and mean ± 1SEM across participants (*n* = 6) is shown as white circles with error bars. Per-participant data on filter use are given in Supplementary Fig. 1.

Light exposure at even moderate intensities during the night shifts circadian rhythms in physiology and behaviour and attenuates the production of the hormone melatonin.^1^^,^^2^ Light acts as a *zeitgeber*, enabling entrainment of the circadian clock to the periodic changes in ambient light levels.^3^ Generally, brighter light has a stronger *zeitgeber* strength, thus providing a more powerful input drive to the circadian timing system.^3^^,^^4^ These non-visual effects of light on the circadian clock are mediated by the retinohypothalamic pathway, which is largely driven by the intrinsically photosensitive retinal ganglion cells (ipRGCs) expressing the photopigment melanopsin.^5^ The ipRGCs are ‘non-classical’ photoreceptors signalling environmental light intensity independent of the ‘classical’ retinal photoreceptors, the cones and the rods (Fig. 1A and B). The normal trichromatic retina (Fig. 1A) contains three classes of cone photoreceptors—the short [S]-, medium [M]- and long [L]-wavelength sensitive cones—the rod photoreceptors and the ipRGCs. The spectral sensitivities of the underlying photopigments are distinct (Fig. 1B), heavily overlapping, and broadly tuned, with peak spectral sensitivities of ∼420 nm (S cones), ∼530 nm (M cones), ∼558 nm (L cones), ∼500 nm (rods) and ∼480 nm (melanopsin) before filtering of light by the lens and ocular media. The ranges at which these photoreceptors are active differ (Fig. 2A), and together they span a wide range of intensities. Cones respond in moderate to bright light (photopic light levels; absolute threshold^6^^,^^7^ ∼10 log photons cm^−2^ s^−1^). Rods, expressing rhodopsin, on the other hand, are 1000–10 000 times more sensitive and signal in dim and dark light (scotopic light levels; absolute threshold^6^^,^^7^ ∼7 log photons cm^−2^ s^−1^). Importantly, rods saturate at photopic light levels,^8^ making them ill-suited for encoding visual signals at bright light levels. The threshold for ipRGCs is estimated to be higher than that of the cones (absolute threshold^6^^,^^7^ ∼11 log photons cm^−2^ s^−1^).

In *congenital autosomal recessive achromatopsia* (ACHM), also called *rod monochromacy* (estimated prevalence 1 in 30 000–50 000 people^9^), the cone photoreceptors are non- or dysfunctional (Fig. 1C and D). This is due to mutations in the genes *CNGA3*, *CNGB3*, *GNAT2*, *PDE6H* and *PDE6C* which affect different aspects of the phototransduction process in cone cells.^10^ In addition, mutations in *ATF6* have been shown to also cause ACHM. As the cones are sensitive to moderate to bright lights and responsible for vision of colour, motion and spatial details at daylight light levels, patients with congenital ACHM lack functional photoreception in the upper range of typical day light exposures. This leads to strong visual discomfort, glare and light aversion.^11^ Congenital achromats are hypersensitive to light,^12^ with corneal photosensitivity thresholds being 100–1000 times lower than for healthy controls.^13^ This can be partially explained by the saturation of rods, which cannot be mitigated by the modification of pupil size (as this alone can only modify retinal illumination by a factor of ∼16 between minimal and maximal pupil size^14^) To be able to cope with typical, in particular daytime light levels, management of congenital ACHM includes the use of tinted filter glasses.^15^

While aspects of rod-mediated visual function in ACHM have been examined before, the question of non-classical photoreception in congenital ACHM has to our knowledge not yet received scientific attention. The authoritative monograph on vision in congenital ACHM does not contain any discussions on non-visual effects of light,^11^ not least because it predated the discovery of melanopsin. There is, however, anecdotal evidence for an adjustment of the circadian system in congenital achromats: A 1992 *New York Times* article on congenital ACHM stated that ‘[m]any with the disorder are proud night owls, who love going out after dark’,^16^ and a publication by *The Achromatopsia Network* suggests that many achromats prefer timing of outdoor and recreational activities to the ‘magical time of twilight’.^17^

Previously, it has been shown that in some individuals who are functionally blind, the melatonin-suppressive effect of light is preserved due to a functioning melanopsin-based ipRGC system even in the absence of cone and rod function.^18^^,^^19^ Direct evidence for a functional preservation of melanopsin-mediated ipRGC function has also been found in other retinal conditions (e.g. Leber congenital amaurosis^20^). Importantly, however, these individuals do not necessarily experience the severe discomfort reaction to light typical for ACHM and therefore may indeed be exposed to much more daytime light levels than achromats. We hypothesized that the extreme light sensitivity, light avoidance and ensuing use of filters lead to reduced light exposure, which translates into a regular but later chronotype. In this case series, we examined the sleep and circadian phenotype in a group of genetically confirmed congenital achromats [*n* = 7, age range 30–72 years; *CNGB3* (*n* = 5) and *CNGA3* (*n* = 2) genotype], employing a comprehensive suite of self-reporting, actimetry and physiological measurements to arrive at the first picture of how sleep and circadian rhythms are affected by a reduced light exposure at photopic levels, and the lack of a functional cone system.

## Materials and methods

### Participants

We recruited participants through advertisements targeted to ACHM patients via the Achromatopsie Selbsthilfeverein e. V., a self-help organization of achromats, and Retina Suisse. A total of ten (*n* = 10) patients responded to our adverts and agreed to participate. Of these, nine (*n* = 9) patients completed the surveys, six (*n* = 6) completed the observational period and five (*n* = 5) the at-home melatonin assessment. One participant completed the melatonin assessment in the laboratory. Here, we only consider data from the seven (*n* = 7) participants with genetic confirmation of autosomal recessive ACHM (Table 1). All participants underwent remote psychiatric examination by the study physician using the telephone-administered MINI-DIPS-OA,^21^ none revealing clinical psychiatric problems at the time of test. One participant habitually used trimipramine, which is known to affect sleep but has no known effects on the circadian system.

**Table 1:** Genotypes of all participants in this study

Patient	Genotype
*s001*	CNGB3: NM_019098: c.[1148delC];[1255G>T] NP_061971.3: p.[(T383Ifs*13)];[(E419*)]
*s002*	CNGB3: NM_019098: c.[1148delC];[1148del] NP_061971.3: p.[(T383Ifs*13)];[(T383Ifs*13)]
*s003*	CNGB3: NM_019098: c.[1148delC];[1148del] NP_061971.3: p.[(T383Ifs*13)];[(T383Ifs*13)]
*s004*	CNGB3: NM_019098: c.[1148delC];[1148del] NP_061971.3: p.[(T383Ifs*13)];[(T383Ifs*13)]
*s005*	CNGA3: NM_001298: c.[458C>T; 1585 G > A];[1228C>T] NP_001289.1: p.[(T153M);(V529M)];[(R410W)]
*s006*	CNGB3: NM_019098: c.[1148delC];[1304C>T] NP_061971.3: p.[(T383Ifs*13)];[(S435F)]
*s007*	CNGA3: NM_001298: c.[830G>A];[1706G>A] NP_001289.1: p.[(R277H)];[(R569H)]

### Saliva and melatonin assays

Participants collected saliva every 30 min from 5 h before to 1 h after their habitual bedtime. Samples were refrigerated and shipped to us for biochemical assays (radioimmunoassay for melatonin). Saliva samples were collected at home (*n* = 5) and in the laboratory (*n* = 1) using Sarstedt salivettes (Sarstedt AG, Sevelen, Switzerland). Following the *Sleep Check* protocol (Bühlmann Laboratories AG, Allschwil, Switzerland^22^^,^^23^), participants received written instructions to avoid exposure to bright light (dim light from a reading light and television was allowed), to not eat during the collection period and not eat bananas and chocolate in the day of collection, to not consume drinks containing artificial colourants, caffeine (e.g. coffee, black, green and ice tea, cola) and alcohol, and avoid intake of medications containing aspirin or ibuprofen. Participants were instructed to rinse their mouths 15 min prior to sample collection, leave the salivette swab in their mouths for 3–5 min, and not handle the swab with their hands. Upon extraction of the swab, they were instructed to refrigerate the samples immediately, and ship them using express shipping methods without further cooling as soon as possible (typical transit time estimated between 1 and 4 days). We again followed the *Sleep Check* protocol here, which suggests refrigerating (rather than freezing) samples. After arrival in the laboratory, the samples were centrifuged and frozen at −20°. These were then either transferred for analysis to the local laboratory (*n* = 1 participant) or shipped on dry ice to Groningen (Chrono@Work, Groningen, Netherlands; *n* = 5 participants), for determination of melatonin concentrations using a direct double-antibody radioimmunoassay (RK-DSM 2 RIA; Bühlmann Laboratories AG, Allschwil, Switzerland), with detection limit (LoB) 0.3 ± 0.21 pg/ml (*n* = 13). The intra-assay coefficients of variation were 10.1% for at 2.5 ± 0.2 pg/ml (*n* = 15) and 13.3% at 23.4 ± 3.1 pg/ml (*n* = 15). The inter-assay coefficients of variation were 15.4% at 2.4 ± 0.4 pg/ml (*n* = 15) and 10.6% at 24.1 ± 2.5 pg/ml (*n* = 15). The evening melatonin profile was fitted with a piecewise linear-parabolic function using the interactive computer-based hockey-stick algorithm to calculate the individual melatonin onset (v2.4).^24^

### Genetic confirmation

All participants were genetically confirmed achromats (Table 1). Five of these were *CNGB3*-associated ACHM patients, while two of them carried mutations in the *CNGA3* gene. Of the six participants who participated in the observational study and the melatonin assessment, five were *CNGB3*-ACHM patients and one was a *CNGA3*-ACHM patient. Genetic confirmation in a research setting was performed by the Institute for Ophthalmic Research, Centre for Ophthalmology, University of Tübingen, Germany.

### Surveys

All survey data were collected and managed using REDCap electronic data capture tools hosted at the University of Basel. Patients completed the Pittsburgh Sleep Quality Index,^25^ the Epworth Sleepiness Scale,^26^ the Morningness–Eveningness Questionnaire,^27^ the Munich Chronotype Questionnaire,^28^ the NEI Visual Function Questionnaire (25 items)^29^ and the Visual Light Sensitivity Questionnaire-8.^30^

Participants also completed custom visual discomfort and light sensitivity, light avoidance and filter use questionnaires. All three questionnaires used commonly encountered lighting conditions and asked for ratings of visual discomfort without filters, light avoidance without filters, as well as frequency of filter use under these conditions using a 5-item Likert scale. The lighting conditions included were direct sunlight, daylight (clear sky without direct sunlight), daylight (cloudy), sunrise and sunset, and twilight (outdoor category), bright, moderate and dim indoor illumination (indoor category), and smartphones, TV and computer use. Two participants completed this questionnaire over the telephone.

### Actigraphy and sleep diary

Participants wore a Condor ActTrust (Condor, São Paolo, Brasil) actiwatch over the course of the 21-day observational protocol. We restricted our analysis to the time period from 12:00 (midnight) on Day 2 to 12:00 (midnight) on Day 20. We analysed actimetry data reported in the normalized Proportional Integration Mode as follows: We estimated the periodicity of the actimetry data using the Lomb-Scargle periodogram using MATLAB's plomb function (Mathworks, Natick, MA). Furthermore, to visualize the periodicity, we fit a sum-of-sinusoids to the time bin-averaged (30-min bins) and *z*-scored data with non-linear least squares using MATLAB’s Curve Fitting Toolbox. We incorporated the fundamental frequency (corresponding to a period length of 24 h) and the second harmonic (corresponding to a period length of 12 h). To address nonstationarities in the rhythm which would be masked by bin-averaging and not captured by the Lomb-Scargle periodogram, we also performed a wavelet analysis^31^^,^^32^ on the activity data. Additionally, we implemented standard non-parametric analyses of actigraphy-derived activity cycles^33^ using the pyActigraphy package,^34^^,^^35^ calculating intra-daily stability and intra-daily variability. In addition to wearing an actiwatch, participants completed paper-and-pen sleep diaries during the 21-day protocol, asking for self-reported sleep time and wake-up time.

### Ethical approval

Ethical approval for this study was granted from the Ethikkommision Nordwest- und Zentralschweiz (EKNZ), no. 2018-02335. Genotyping in a research setting was approved by the ethics committee of the University of Tübingen, no. 116/2015BO2. All participants gave informed consent.

### Data and code availability

All code and data underlying this article is available on a public GitHub repository (https://github.com/spitschan/Spitschan2021_Brain_Communications).

## Results

### Congenital achromats experience altered light exposure due to sensitivity to light

We confirmed elevated light sensitivity in the sample of congenital achromats, finding high sensitivity to bright lighting conditions, such as direct sunlight, daylight under a clear sky, as well as bright indoor illumination (Fig. 2A). This sensitivity to light translates to higher degrees of avoidance of exposure to bright light (Fig. 2B). In one patient (s006), we confirmed light sensitivity and retained pupil responses to light^36^ in an in-laboratory protocol (Supplementary Fig. 3).

This sensitivity to light is typically managed using optical filters^37^ integrated in spectacles or contact lenses. These filters reduce the activation of rods and thereby alleviate visual discomfort and prevent saturation of the rods.^38^ In Germany, where six of seven of our patients were residing, filters with a transmittance ≤75% and long-pass cut-off filters (cut-off wavelength >500 nm) are prescribable by federal regulation^39^ and therefore can be reimbursed through health insurance. In practice, many congenital achromats have at least two filter glasses, a cut-off filter (such as a Zeiss F540, 50% absorption at 540 nm) for indoor use and a cut-off filter with an additional tint (such as Zeiss F90, 90% absorption at 600 nm). In our sample of congenital achromats, we characterized the habitual use of filters using a questionnaire (Supplementary Fig. 1). All participants used a very strong filter to reduce retinal illumination in bright outdoors conditions (Supplementary Fig. 1). Some of our patients (s003 and s005) use up to five separate filters under different conditions, highlighting the complex requirements for management of congenital ACHM, as well as individual differences in light sensitivity.

Owing to the substantial overlap of rods and ipRGCs in their response to different wavelengths (Fig. 1D), we hypothesized that filter use to reduce rod activation would also reduce ipRGC activation. We tested this hypothesis by examining how spectral filters prescribed in congenital ACHM change the signals of rods and ipRGCs (Supplementary Fig. 2b). As predicted from the strong overlap and correlation of rod and melanopsin spectral sensitivities, we confirm that rod and ipRGC signals are strongly correlated in everyday light exposures (Supplementary Fig. 2a). We examined the change of rod and ipRGC signals by simulating the world seen through two common spectral filters (F540 and F90). We find that these two filters reduce the activation of rod and ipRGC signals by a factor of ∼0.1× (F540) and ∼0.01× (F90) on average, respectively (Supplementary Fig. 2c). In sum, the use of filters to manage severe visual discomfort in congenital ACHM leads to a significant reduction in habitual light exposure and therefore a significant change in chronic ipRGC activation, the photic driver of the circadian system.

### Survey-estimated chronotype and sleep

All results for the Pittsburgh Sleep Quality Index (PSQI), Epworth Sleepiness Scale (ESS), Morningness-Eveningness Questionnaire (MEQ), Munich Chronotype Questionnaire (MCTQ), NEI Visual Function Questionnaire-25 (NEI-VFAQ-25) and Visual Light Sensitivity Questionnaire-8 (VLSQ-8) are listed in Table 2. Unexpectedly, we found low scores on the NEI Visual Function Questionnaire-25 [composite score median ± interquartile range (IQR) 33.15 ± 4], indicating low vision-related Quality of Life, and high sensitivity to light on the Visual Light Sensitivity Questionnaire-8 (median ± IQR: 27 ± 4). For the Pittsburgh Sleep Quality Index, assessing sleep quality over the previous four weeks, we found a range of 3–12 (median ± IQR: 7 ± 3.5) with 6 above usual cut-off of 5, indicating low sleep-quality. However, only one participant was found to have excessive daytime sleepiness according to the Epworth Sleepiness Scale (median ± IQR: 6 ± 3). We found a range of Morningness–Eveningness Questionnaire values between 24 and 49 (median±IQR: 40 ± 13), with one definitive evening type, two moderate evening types, and three neutral types. Using the Munich Chronotype Questionnaire, we found a median ± IQR mid-sleep MSF_sc_ (mid-sleep on free days) on ∼4:00 ± 0.55, corresponding to intermediate/slightly late chronotypes.^40^ In aggregate, the survey instruments indicate a slight nominal tendency to late chronotypes.

**Table 2:** Demographic details, survey results and participation in sub-studies

Patient	Age	Sex	Sleep		Chronotype		Visual function		Participation			
			PSQI^25^	ESS^26^	MEQ^27^	MSF_sc_^28^	NEI-VFQ-25 (Composite Score)^29^	VLSQ-8 total^30^	Filter use survey	Sleep and chronotype survey	At-home DLMO assessment	Laboratory DLMO assessment
*s001*	32	F	9	6	44	4.50	33.15	31	✓	✓	✓	
*s002*	64	F	3	2	40	3.99	32.83	19	✓	✓	✓	
*s003*	41	F	12	6	46	3.60	36.67	27	✓	✓	✓	
*s004*	54	F	7	9	49	4.09	27.00	28	✓	✓	✓	
*s005*	72	F	7	8	24	3.88	35.19	26	✓	✓	✓	
*s006*	66	M	5	14	31	5.00	33.61	26	✓	✓		✓
*s007*	40	F	10	6	33	2.00	27.96	31		✓		
Median			**7**	**6**	**40**	**3.99**	**33.15**	**27**	*n* = 6	*n* = 7	*n* = 5	*n* = 1
IQR			**4**	**3**	**13**	**0.56**	**4.00**	**4**				

DLMO, dim-light melatonin onset; ESS, Epworth Sleepiness Scale; IQR, interquartile range; MEQ, Morningness–Eveningness Questionnaire; NEI-VFQ, NEI Visual Function Questionnaire; PSQI, Pittsburgh Sleep Quality Index; VLSQ-8, Visual Light Sensitivity Questionnaire-8.

### Congenital achromats have regular rest-activity cycles

**Figure 3:**
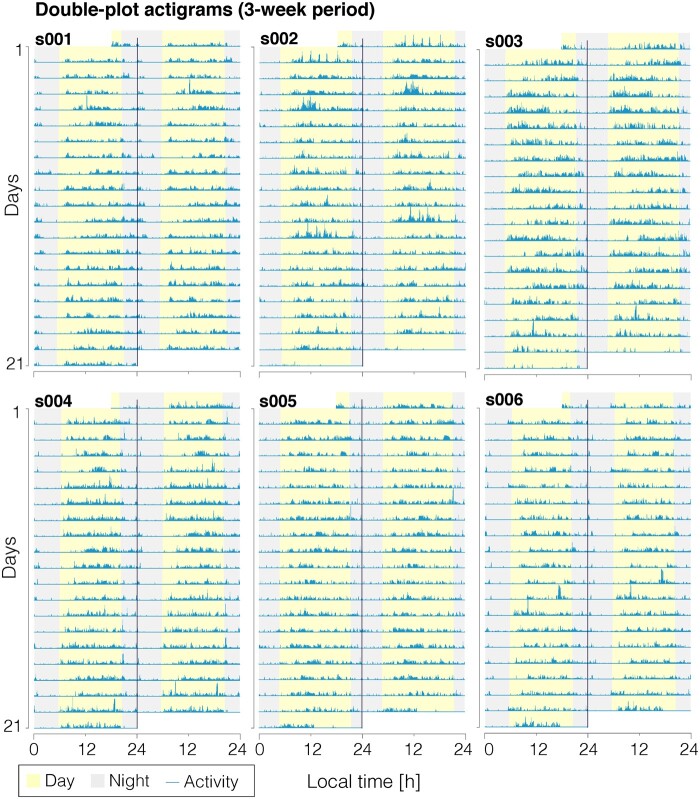
**Wrist-referenced actigraphy shows regularity in activity in a group of six achromats (*n* = 6)**. Data shown across the three weeks of observation. Participants were not instructed to follow a particular rest-activity pattern. Actigrams are shown as double-plots with the *x*-axis spanning a period of two consecutive days. Shading for day and night is taken from sunrise and sunset times at these chronological dates.

**Figure 4:**
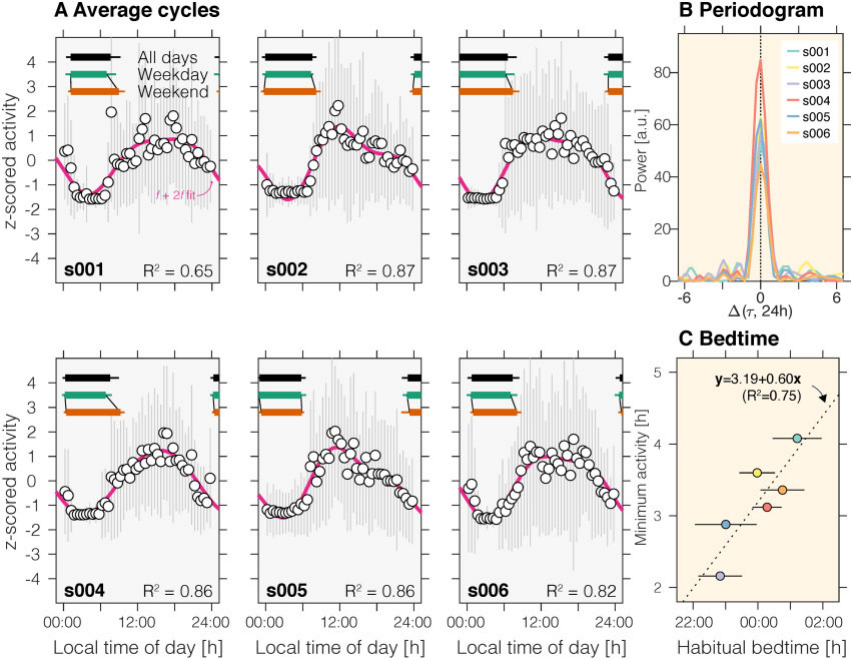
**Actigraphy-derived analyses.** (**A**) Data from the 21-day observational period were collapsed across days within time of day to yield the average time-of-day activity curves (60 min bins). For visualization, averaged data were fitted using a sine–cosine *f* + 2*f* fit, where *f* = 1/(24.0 h). All participants show a strong diurnal activity rhythm which can be characterized by a sinusoidal fit on the averaged values (range of *R*^2^ values: 0.65–0.87). *Insets*: Average (mean ± 1 SD, horizontal error bars shown on one side only) bed and wake-up times across the 21-day observational period across all days (black), or aggregated by weekday (green; Monday–Friday), and by weekend (red; Saturday and Sunday). (**B**) Periodogram analysis of actigraphy data, showing a 24-h period, confirmed by a wavelet analysis in Supplementary Fig. **4**). (**C**) Relationship between habitual bed time as well as the actigraphy-derived minimum activity timing (obtained from minimum of sinusoidal fit).

Of our seven participants, six participants completed a three-week long assessment during which they wore actigraphy watches and completed a sleep diary but were not instructed to follow any particular sleep–wake schedule. We found regular rest-activity cycles in all individuals (Fig. 3; wrist-referenced light measurements in Supplementary Fig. 5). We subjected the actigraphy data to a Lomb-Scargle periodogram analysis (Fig. 4B), finding that the rest-activity patterns are periodic with a period length of 24 h. To examine possible non-stationarities in the rhythm that would not be captured using the periodogram analysis, we confirmed the 24-h periodicity using a wavelet-based analysis (Supplementary Fig. 4). The probability that this 24-h periodicity is due to participants having a free-running rhythm with 24-h period is very unlikely (*P* < 0.0001 assuming an exact period of 24 h, and *P* = 0.018 for a range of periods in the interval 24 ± 0.2 h; Supplementary Fig. 6). Additionally, we assessed the regularity and fragmentation of the participants’ activity rhythms.^33^ The regularity (intra-daily stability: 0.65 ± 0.05 [mean + 1SD]) is slightly lower than the estimated population average^41^ but significantly higher than a previously characterized sample of psychiatric patients with sleep–wake problems.^42^ Fragmentation (intra-daily variability: 0.80 ± 0.02), i.e. the frequency and extent of transition between periods of low and high activity, higher than in the estimated population average,^41^ confirming the lower self-reported sleep quality above the normal cut-off.

We further examined whether the sleep–wake rhythms of the congenital achromats exhibit social jetlag,^43^ which is the phenomenon that participants go to bed and wake up later on the weekends. By drawing on self-reported bed and wake-up times, we found that most of our participants showed on average a delay in their weekend wake-up times (Fig. 4) of approximately 1 h difference in wake-up time on the weekend (median ± IQR: 1.12 ± 1.26), but a smaller nominal difference in bed-time (median ± IQR: 0.17 ± 0.30).

### Congenital achromats have normal melatonin secretion profiles and phase angles of entrainment

**Figure 5:**
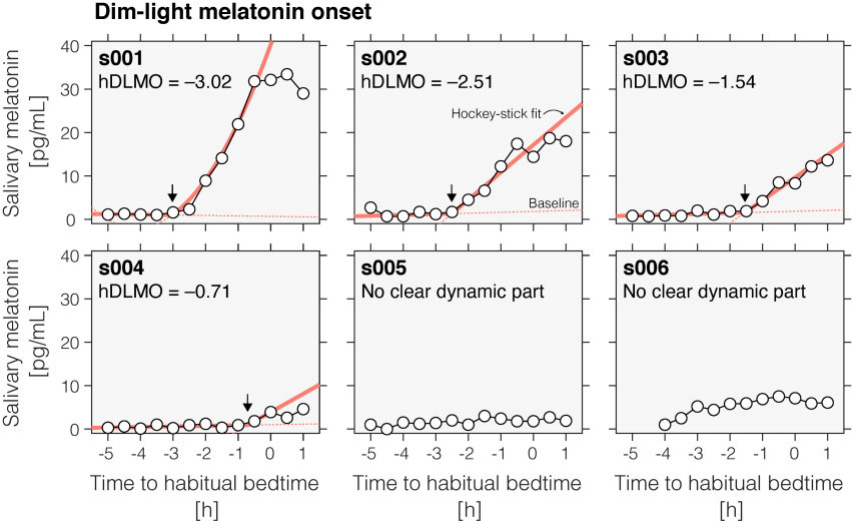
**Normal melatonin phase angles of entrainment in four congenital achromats (*n* = 4; total of 6 tested). Dim-light melatonin onset profiles as a function of habitual bedtime, assessed in an at-home measurement protocol using saliva collection. Saliva samples were assayed using radioimmunoassay (see details in text) and DLMO timing was extracted using Hockey Stick software.**^24^ Two of the six participants did not show a clear dynamic rise in their melatonin profiles.

While our actigraphy results strongly point to preserved normal diurnal rhythms in activity and behaviour with a period of 24 h, these data themselves do not establish that this is due to a preserved circadian rhythm. For example, it is conceivable that the behavioural entrainment can be attributed to non-photic *zeitgebers* (e.g. alarm clocks as a simple example). To rule out this possibility, we examined the secretion of melatonin during the evening hours in a modified at-home dim-light melatonin onset (DLMO) protocol. In four of six participants who participated in this study, we found a clear rise in melatonin levels (Fig. 5). The phase angle of entrainment ranged between ∼3 h to ∼45 min prior to habitual bedtime. This distribution is within the normal range for the melatonin phase angle of entrainment.^44^ In two participants (s005 and s006; Fig. 5) corresponding to the two oldest patients in our study which habitually take beta-blockers, we failed to detect an increase in melatonin levels in the evening in the expected range relative to habitual bedtime.

## Discussion

In the first investigation of sleep and circadian phenotype in ACHM, we find that congenital achromats have both normal rest-activity cycles (6/6 participants) and phase angles of entrainment (4/6 participants) in the absence of a functional cone system, adding to our understanding of how light and alterations in the retinal photoreceptors affect circadian and sleep physiology. Importantly, our results show that a functional cone system may not be necessary for normally entrained circadian melatonin and sleep–wake cycles in congenital achromats. We also confirm the findings of previous studies, which have found much lower light discomfort thresholds for congenital achromats than for health controls (∼3 vs. ∼1500 lux),^13^ in our sample of patients.

In this case series, we examined patients with congenital ACHM solely based on genotype (*CNGB3: n* = 5; *CNGA*3: *n* = 2) without any further extensive testing of phenotype and formal visual function testing. We cannot exclude that our patients may have residual cone function. Yet, from the existing data on the functional assessment of CNGA3 missense variants, we believe that the two included *CNGA3*-ACHM patients are complete achromats. The p. V529M, p. R410W, p. R277H and p. R569H missense mutations have been shown to result in complete loss of channel function in prior literature on functional testing of these variants.^45^^,^^46^ Regarding the *CNGB3* genotypes, the c.1148delC mutation leads to a shift of the open reading frame and a premature termination codon, resulting in loss of CNGB3. It is therefore expected to be a null allele. The p. S435F mutation is the Pingelapese colour blindness mutation,^47^^,^^48^ but it has also been found in European patients. The patients were sent to us with a clinical diagnosis of ‘achromatopsia’, making the diagnosis of ‘incomplete achromatopsia’ unlikely. In heterologous expression in Xenopus oocytes,^49^ the p. S435F mutation does lead to residual channel function of the mutant CNGA3 homotetrameric channel, but how this translates to cone function is speculation. Future research should consider more extensive visual function testing to determine the exact retinal phenotype and its characteristics, including possible residual cone function testing and possible alterations of rod signals,^50–52^ on a per-participant basis, and how these may influence or explain our results obtained herein.

In two participants (s005 and s006), the oldest in our sample, we failed to find a DLMO. While this might be due to mistiming of the saliva collection protocol, both individuals had normal rest-activity cycles, suggesting that the lack of measurable DLMO in these participants may be due to the well-known interaction of beta-blockers with melatonin secretion^53^ or due to overall reduced melatonin production in these participants.

In people with a trichromatic retina, entrainment to a 24-h cycle (but not lower or higher period lengths) can be supported by dim light at around 1.5 lux under very tightly and explicitly controlled conditions,^54^ and typical indoor light levels.^3^ Similarly, some laboratory studies performed with pharmacological pupil dilation and dark adaptation have also found very low melatonin suppression thresholds.^55^ The overwhelming evidence suggests that moderate light exposures are necessary to produce an appreciable effect in circadian entrainment^56^^,^^57^ and melatonin suppression. Outdoor light exposure is also systematically related to chronotype,^58^ with higher outdoor light exposure leading to phase-advanced activity cycles.^59^ This is consistent with our findings that congenital achromats show a tendency to later chronotypes, likely due to their lack of a strong light exposure signal.

Whether rod or cone signals alone are sufficient to influence human circadian and neuroendocrine responses to light in humans is currently not known and will require further investigation. There is indirect evidence for rod and cone participation in non-visual responses,^60–62^ which may be time-dependent.^63^^,^^64^ Participants with colour vision deficiencies affecting the L (protanopia) or M (deuteranopia) cones show normal melatonin suppression responses to light, indicating that neither class is necessary for melatonin suppression.^65^ In animal models, rods have been found to contribute to phase shifting responses,^66^ thereby effectively extending the range at which light can contribute to circadian photoentrainment.

Retinal irradiance is modulated by pupil size, which is primarily controlled by melanopsin, though in principle all photoreceptors can contribute to its control.^67^^,^^68^ Congenital achromats retain pupil constrictions to light,^36^ which we have confirmed here in one patient. As the important biological variable for non-visual responses to light is retinal irradiance (rather than corneal irradiance),^69–71^ determining the actual light exposure requires factoring in the pupil size. Future investigations should consider the conjoint measurement of pupil size and near-corneal irradiance (and spectral filtering by filters), to determine effective physiologically-relevant light exposure in congenital achromats.

Behavioural light avoidance and use of filters that reduce retinal illuminance leads to the chronic modification of the ‘spectral diet’ of congenital achromats. An adaptation mechanism may tune the sensitivity of circadian photoreception to the range of available light intensities in the environment. In trichromatic observers, chronic modification of retinal input through the use of blue-filtering contact lenses over a two-week period^72^ or through the natural ageing^73^ leads to an adaptation of the melatonin-suppressive response to light. A similar mechanism could be at play in congenital ACHM, indicating a flexible gain control mechanism that normalizes the sensitivity of the circadian system to the range of habitual retinal illuminances. This hypothesis deserves further empirical testing.

The light–dark cycle is the primary driver of circadian entrainment,^74^ but the circadian system is also sensitive to nonphotic zeitgebers,^75^ including physical exercise,^76–80^ meal times,^81^^,^^82^ auditory stimul^83^ and social cues. We did not assess these non-photic cues in this study. Future investigations should include either assessment of these cues in field conditions, and focus on controlled, constant-routine protocols performed in the laboratory.^84^ Under all circumstances, congenital achromats do experience the light–dark cycle, albeit in an altered way and reduced in amplitude. While meal timing can affect the circadian clock,^82^ these effects may be limited to peripheral oscillators and may not affect DLMO timing.^81^ A promising direction for future research lies in adaptation of circadian photoreception to the reduced range of light intensities. Importantly, the results presented here may apply to other inherited or progressive retinal disorders and diseases characterized by hypersensitivity to light.

## Supplementary material

Supplementary material is available at *Brain Communications* online.

## Supplementary Material

fcab159_Supplementary_Data

## Abbreviations

ACHM =: congenital autosomal recessive achromatopsia
DLMO =: dim-light melatonin onset
ipRGCs =: intrinsically photosensitive retinal ganglion cells
IQR =: interquartile range
L cones =: long-wavelength-sensitive cones
M cones =: medium-wavelength-sensitive cones
S cones =: short-wavelength-sensitive cones
