# Supplementary material for: Sleep and circadian phenotype in people without cone-mediated vision: a case series of five CNGB3 and two CNGA3 patients
Source: Brain Commun. 2021 Jul 18;3(3):fcab159. doi: 10.1093/braincomms/fcab159 (PMC8385249; doi:10.1093/braincomms/fcab159)
Supplement: fcab159_Supplementary_Data [file fcab159_supplementary_data.pdf]

# Sleep and circadian phenotype in people without cone-mediated vision: case series of five *CNGB3* and two *CNGA3* patients

Manuel Spitschan[1, 2, 3, ¶, [0000-0002-8572-9268]], Corrado Garbazza[2, 3, [0000-0002-8606-2944]], Susanne Kohl[4, [0000-0002-6438-6331]], & Christian Cajochen[2, 3, [0000-0003-2699-7171]]

[1] *Department of Experimental Psychology, University of Oxford*, United Kingdom

[2] *Centre for Chronobiology, Psychiatric Hospital of the University of Basel*, Switzerland

[3] *Transfaculty Research Platform Molecular and Cognitive Neurosciences, University of Basel,* Switzerland

[4] *Institute for Ophthalmic Research, Centre for Ophthalmology, University of Tübingen,* Germany

[¶] To whom correspondence should be addressed: Dr Manuel Spitschan, Email: manuel.spitschan@psy.ox.ac.uk

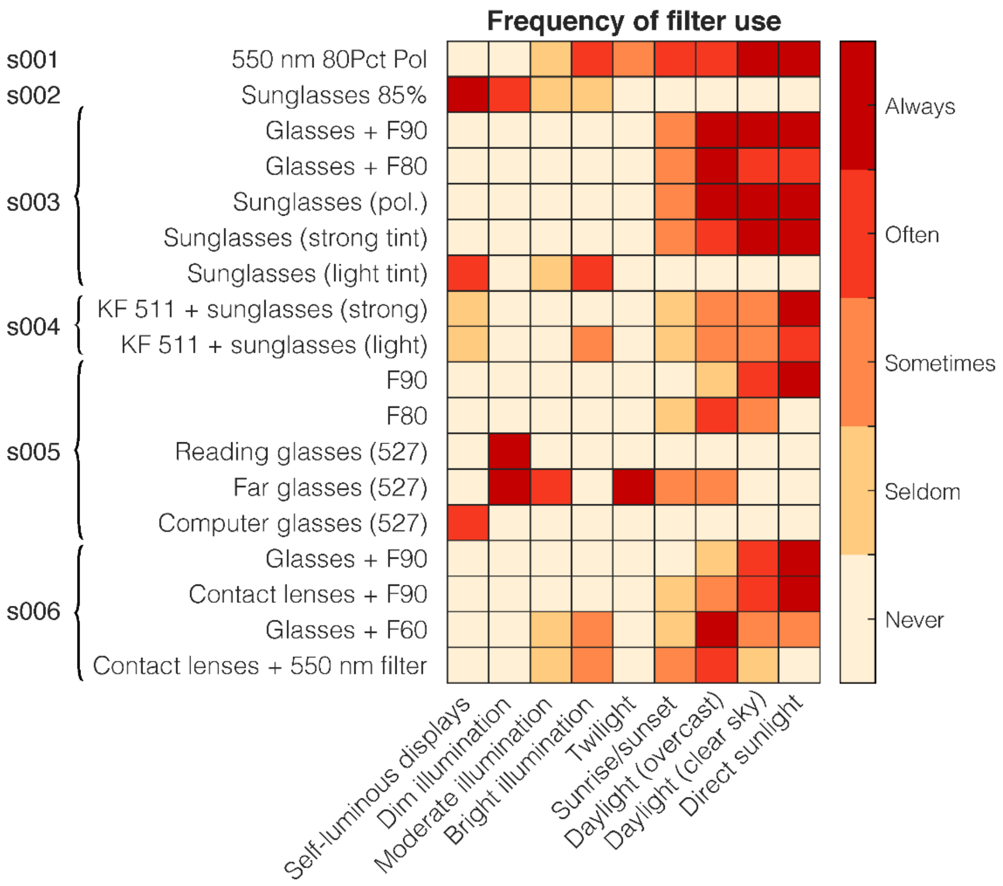


**Supplementary Figure 1.** ***Habitual filter use.*** Participants were asked to indicate the frequency of filter use under a range of commonly encountered lighting conditions using a 5-item Likert scale. The number of filters used varied widely between participants (1-5 filters used), though all used some filters under daylight intensities.

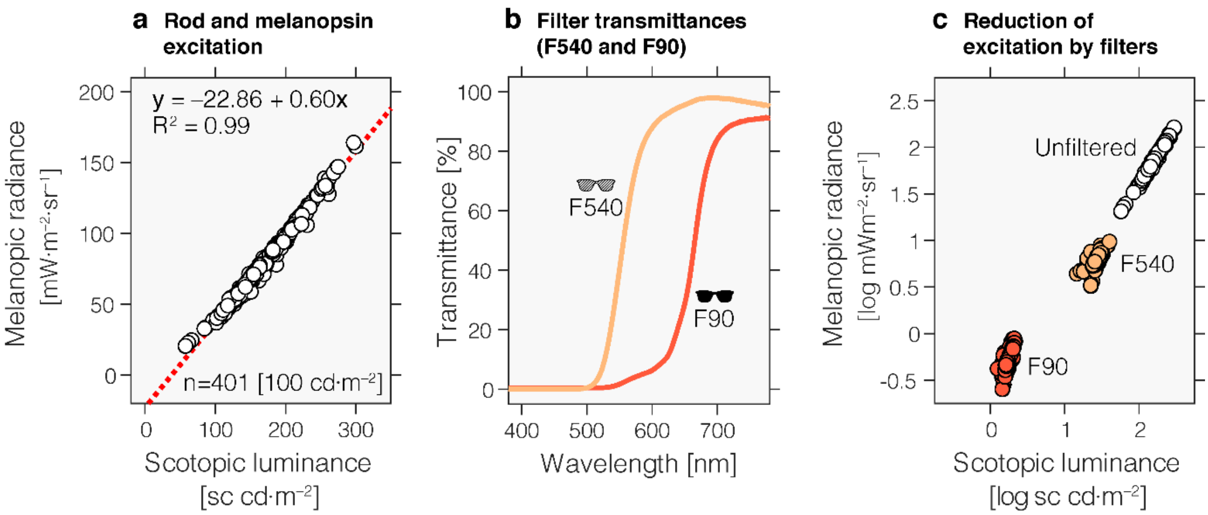


**Supplementary Figure 2. *Spectral filters affect rod and melanopsin signals in the achromatic retina. a*** Simulation of rod and melanopsin-expressing ipRGC signals under wide range of spectral conditions. We simulated the distribution of rod signals (expressed as scotopic luminance) and ipRGC signals (expressed as melanopic radiance) while pegging the photopic luminance to match 100 cd/m$^2$ for 401 spectra representing a wide variety of light sources, including daylight, fluorescent and LED light [1]. Under these conditions, rod and melanopsin expressing ipRGC signals are highly correlated and linear with each other, not least owing to the small spectral separation of the rod and ipRGC spectral sensitivities (correlation of their spectral sensitivities: Pearson's $r$=0.946, p<0.001). Melanopic and scotopic quantities were calculated by matrix-multiplying the spectra with the spectral sensitivities contained in the CIE S 026/E:2018 standard [2]. Rod responses were scaled by 1700 lm·W$^{-1}$ [3]. ***b*** Transmittances of F540 and F90 tinted filter glasses, which are commonly prescribed in congenital ACHM. The spectral transmittances of the patient-owned F540 and F90 filters were measured between 250 and 2500 nm at 1 nm resolution using a Varian Cary 500 Scan UV-Vis NIR Spectrophotometer (Varian Inc., Palo Alto, CA). ***c*** Simulation of rod and ipRGC signals under the two filters (F90 and F540). The filters reduce both rod and ipRGC radiance by approximately 1 and 2 log units, on average. We confirmed these theoretical calculations using an independent dataset of personalized light exposure measurements with spectral resolution (n=1 healthy control participant; n=4213 usable spectra between 1 and 10,000 lx; clip-on nanoLambda Spectrometer, Daejeon, Korea). For the F540 filter, rod responses are on average reduced by a factor of 0.16× ≈ 0.8 $\log_{10}$ units, and melanopic responses are reduced by a factor of 0.08× ≈ 1.1 $\log_{10}$ units. On average, The F90 filter reduces rod responses by a factor of 0.01× ≈ 1.98 $\log_{10}$ units, and melanopic responses are reduced by a factor of 0.007× ≈ 2.16 $\log_{10}$ units,

confirming that everyday light exposure profiles yield a reduced melanopic signal with filter usage.

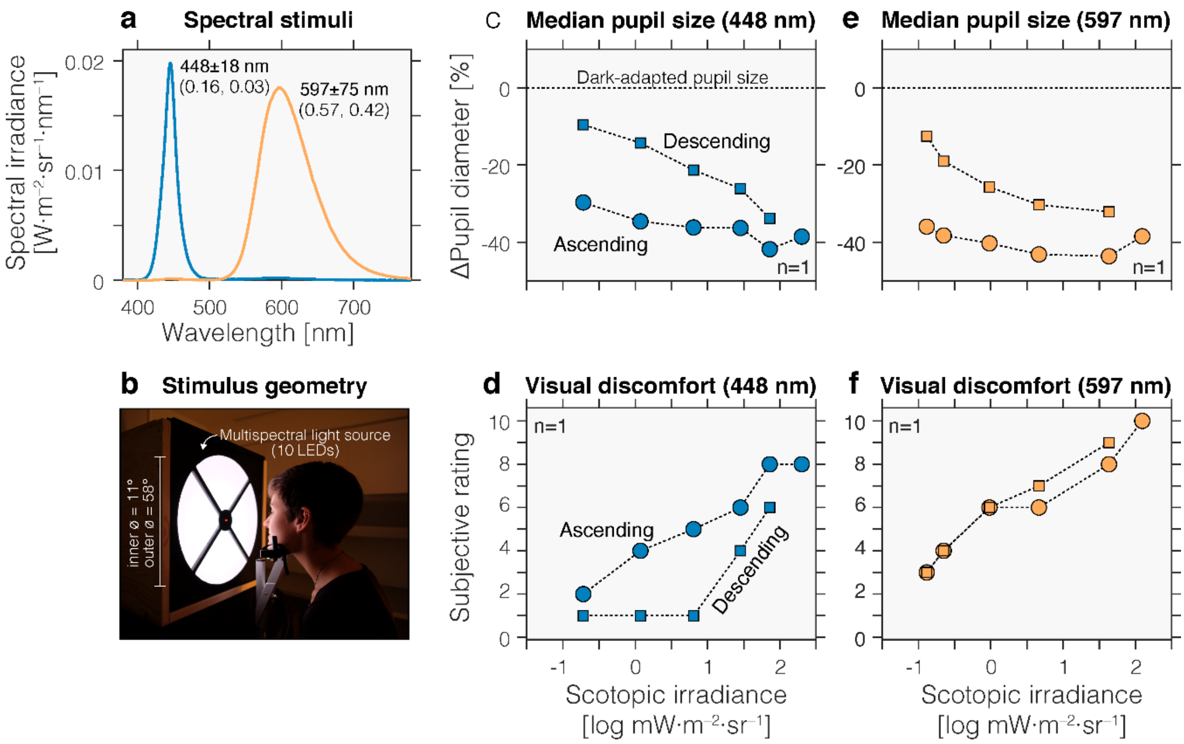


**Supplementary Figure 3.** ***Visual discomfort ratings and pupil responses in a congenital achromat (n=1).*** After adapting to the dark for 20 minutes, the participant viewed wide-field (inner diameter: 11°, outer diameter: 58°) stimuli produced using a 10-primary multispectral light source (LEDmotive SpectraTune Lab, LEDmotive, Barcelona, Spain). 30 seconds. Pupil diameters were extracted using the 2D pixel diameters produced using the Pupil Labs analysis software (Pupil Analysis, https://github.com/pupil-labs/pupil) and summarised using the median over valid data points (after excluding blinks). ***a*** Irradiance spectra of stimuli used (measured using JETI spectraval, JETI Technische Instrumente, Jena, Germany), corresponding to a blue appearing and orange appearing light (to a trichromat). ***b*** Overview of stimulus geometry. Light emitted from a 10-primary tuneable LED-based light source was back-projected on a plexiglass surface, which the participant viewed in free-viewing conditions. ***c*** Pupil responses (relative to dark-adapted pupil size; 20 min dark adaptation period) in response to a series of ascending 448 nm stimuli, increasing in irradiance (squares), or to a series of descending light stimuli, decreasing in irradiance (circles). ***d*** Visual discomfort ratings (n=2 per light level) in response to a series of ascending 448 nm stimuli, increasing in irradiance (squares), or to a series of descending light stimuli, decreasing in irradiance (circles). ***e*** Pupil responses (relative to dark-adapted pupil size; 20 min dark adaptation period) in response to a series of ascending 597 nm stimuli, increasing in irradiance (squares), or to a series of descending light stimuli, decreasing in irradiance (circles). ***f*** Visual discomfort ratings (n=2 per light level) in response to a series of ascending 597 nm stimuli, increasing in irradiance

(squares), or to a series of descending light stimuli, decreasing in irradiance (circles). Differences between ascending and descending pupillary responses are likely due to well-known asymmetries in the acute constriction upon light onset, and acute dilation upon light offset in the pupil.

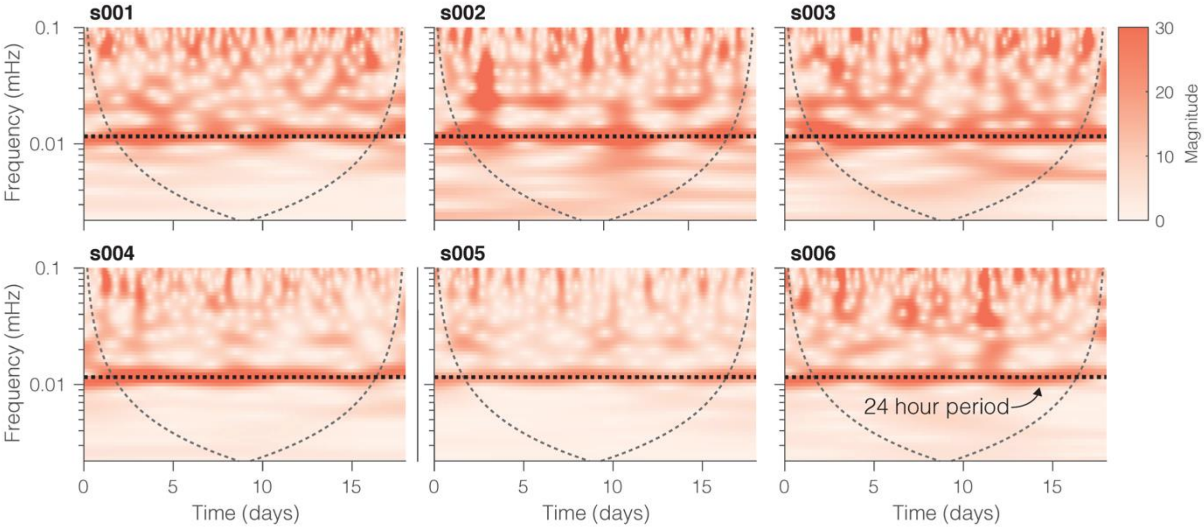


**Supplementary Figure 4: Analysis of actigraphy data accounting for non-stationarity confirms rest-activity cycles with 24-hour period.** To understand the 24-hour periodicity of the measurements in the presence of possible non-stationarities (i.e. changes of phase and period throughout the protocol), actigraphy from all participants (n=6) were analysed using wavelets [4,5]. Activity data (PIMn) were subjected to the continuous wavelet transform using the analytic Morse wavelet with parameters $\gamma = 3$ (symmetry) and $p^2 = 60$ (time-bandwidth product) implemented in MATLAB's cwt function [6,7]. The black dashed line corresponds to the frequency associated to a 24-hour period. Any points outside of the grey dashed 'U'-shaped curve are susceptible to edge effects.

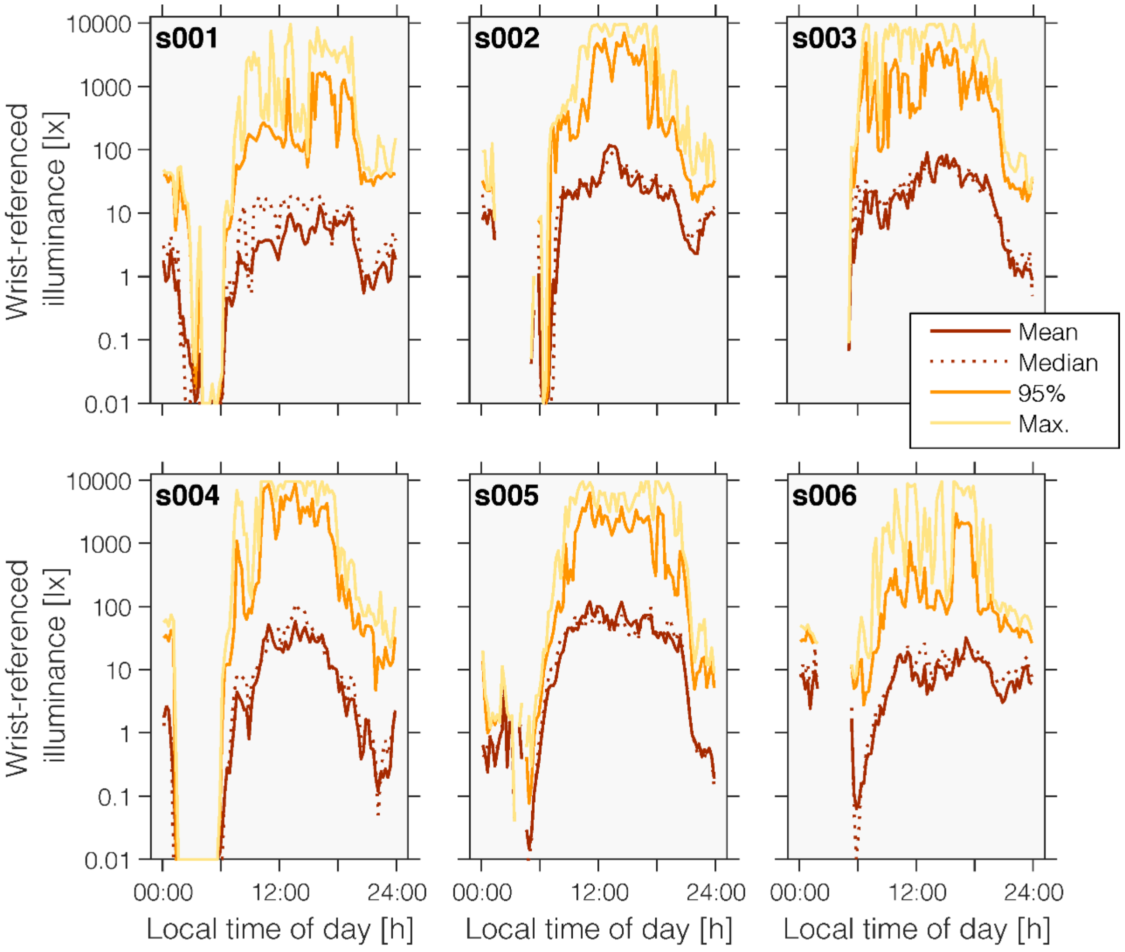


**Supplementary Figure 5:** ***Actigraphy-derived light measurements. a*** Data from the 21-day observational period were collapsed across days within time of day to yield the average time-of-day activity curves (60 minute bins).

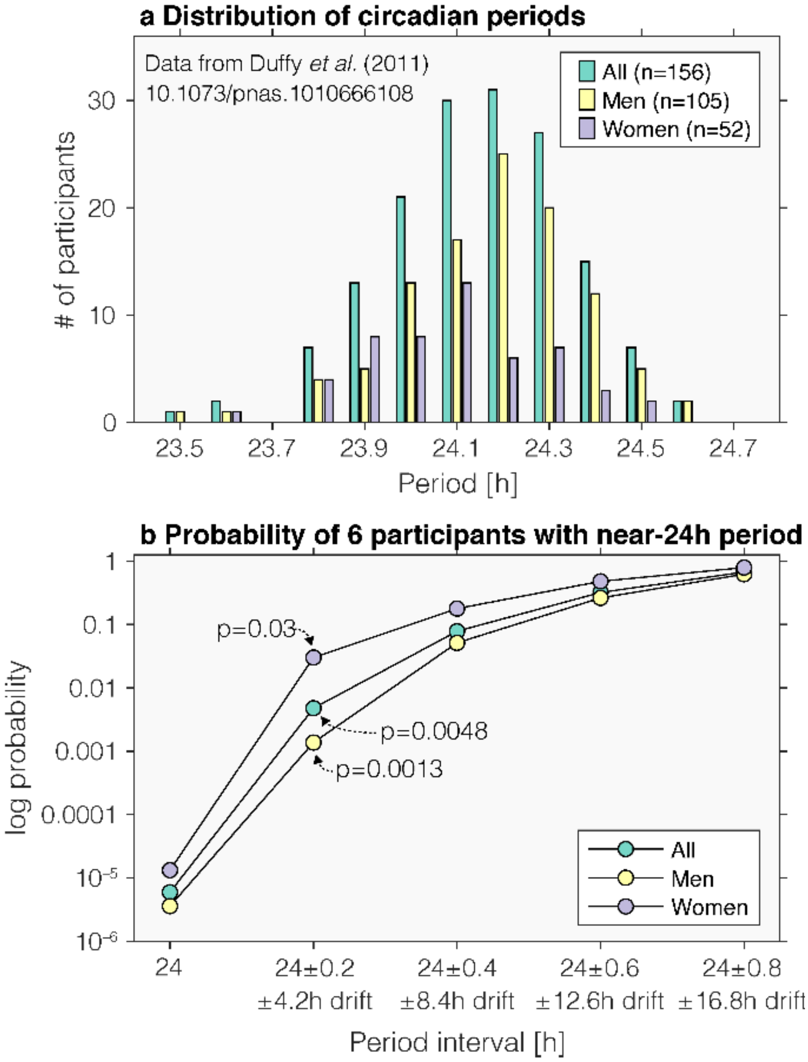


**Supplementary Figure 6: Probability of free-running participants with precise or near-24h period.** ***a*** Distribution of circadian periods derived from temperature measurements in a general population as reported by Duffy *et al.* (2011) in an in-laboratory paradigm. Data are replotted from their Figures 1 (all data; top panel), and Figure 2 (data aggregated by sex). ***b*** Probability to have recruited a total of 6 participants with varying period intervals leading to a range of drift values across an observation epoch of 21 days. The probability to recruit a participant with a period within the 24h "bin" is <0.0001, independent of whether data all data are considered, or disaggregated by sex. Assuming a more liberal criterion, the probability of recruiting a participant with the 24±0.2h bin (leading to a drift of ±4.2h over 21 days) is of course higher, and given by $0.1346^6=0.0048$ for six participants regardless of sex [$0.1538^n=0.03$ for an all-female participant sample of size six, and $0.1238^n=0.0013$ for an all-male sample of size six (arrows indicated in figure)]. We validated these numbers numerically using repeated sampling from the empirical histogram using the inverse transform method and calculating the fraction that all six participants were landed in the period bin 24±0.2h (k=10,000

draws of six participants, m=30 repeats). The probability estimates for sampling six participants in the 24±0.2h bin were 0.0051±0.0007 (both male and female), 0.0301±0.0017 (all female), and 0.0014±0.0004 (all male), respectively. In this experiment, where we had five female and one male participant, we estimate the probability to randomly sample participants with a period between 23.9 and 24.1h to be $0.1538^{5} \times 0.3333 = 0.018$.

**References**


1. Houser KW, Wei M, David A, Krames MR, Shen XS. Review of measures for light-source color rendition and considerations for a two-measure system for characterizing color rendition. *Opt Express.* 2013;21(8):10393-10411.
2. CIE. CIE S 026/E:2018: CIE System for Metrology of Optical Radiation for ipRGC-Influenced Responses to Light. In. Vienna, Austria: CIE Central Bureau; 2018.
3. ISO. *ISO 23539:2005(E)/CIE S 010/E:2004: Photometry —The CIE system of physical photometry.* 2005.
4. Leise TL, Harrington ME. Wavelet-based time series analysis of circadian rhythms. *J Biol Rhythms.* 2011;26(5):454-463.
5. Leise TL. Wavelet analysis of circadian and ultradian behavioral rhythms. *J Circadian Rhythms.* 2013;11(1):5.
6. Olhede SC, Walden AT. Generalized Morse wavelets. *IEEE Transactions on Signal Processing.* 2002;50(11):2661-2670.
7. Lilly JM, Olhede SC. Higher-Order Properties of Analytic Wavelets. *IEEE Transactions on Signal Processing.* 2009;57(1):146-160.
